# Editorial Department

**Published:** 1862-11

**Authors:** 


*The Physician's Hand-Book of Practice, for* 1863. *By* WLLIAM ELMER, M. D. *New York: W. A. Townsend, Publisher, No.* 39 *Walker street,* 1863.

Contents.—Calendar for 1863, Preface, Classification of Diseases, Fevers, Eruptive Fevers, Constitutional Diseases, Diseases of Blood and Blood-Vessels, Diseases of the Brain, Spinal Cord and Nerves, Diseases of Respiratory Organs, Diseases of Circulatory Organs, Diseases of Digestive and Absorbent Systems, Diseases of Internal Organs of Secretion, Cutaneous Diseases, Diseases of Reproductive Organs, Diseases of the Breast, Dr. Marshall Hall's Ready Method, Medicinal Weights and Measures, Abbreviations of Medical Properties of Remedies, List of Remedial Agents, Appendix to the List of Remedies, List of Incompatibles, Balnea Medicata, Remarks on the Writing of Prescriptions, Examples of Extemporaneous Prescriptions, Poisons and their Antidotes, Diagnostic Examination of the Urine, The Pulse—Table of, and other Characters, Explanation of Signs, etc., to be used in the Record, Names and Addresses, Bills and Accounts, Record of Practice and Treatment, Obstetric Calendar, Obstetric Record, Blanks Wants and General Memoranda, Nurses.

This *Hand-Book of Practice* comes to us bound in the highest style of the art, and is an exceedingly attractive pocket companion and memorandum book. It is unsurpassed in its arrangements.

---

*The Physician's Visiting List, Diary, and Book of Engagements, for* 1863. *Philadelphia:* LINDSAY & BLAKISTON, 25 *South Sixth street, above Chestnut.*

Contents.—Almanac, Table of Signs, Marshall Hall's Ready Method in Asphyxia, Poisons and their Antidotes, Table for calculating the period of Utero-Gestation, Blank leaves for Visiting List, do. for Memoranda, etc., etc., do. Addresses of Patients and others, do. Addresses of Nurses, their reference, etc., do. for Accounts asked for, do. for Memoranda of Wants, do. for Obstetric Engagements, do. for Vaccination Engagements, do. for General Memoranda, etc.

The *Physician's Visiting List* is what it claims to be in every respect. It is of convenient pocket size, is plainly but substantially bound, contains exceedingly well arranged blank pages, and is almost a necessity for physicians.

---

*The Physician's Pocket Memorandum, for* 1863. *By* C. H. CLEAVELAND, M. D. *Cincinnati:* Bradley & Webb, Printers.

Contents.—Preface to first edition, Preface to second and third edition, List of Medicines—classification, Class I, Hæmeticæ, Class II, Neuroticæ, Class III, Myonoticæ, Class IV, Adenoticæ, Medicine not Classified, Abbreviations used in Prescriptions, Accidents and Emergencies, Poisons and Antidotes, Post-Mortems, etc., Preservation and Embalming, Prescription of Medicines, Signs used in Record of Practice, Memoranda of Practice, General Memoranda.

The *Physician's Memorandum* has been greatly improved in its present issue. It is of generous size, has a full table of contents, is plainly and substantially bound, and is a good blank book, besides containing much valuable matter.

